# Towards a biological view of multiple sclerosis from early subtle to clinical progression: an expert opinion

**DOI:** 10.1007/s00415-025-12917-4

**Published:** 2025-02-01

**Authors:** Massimo Filippi, Maria Pia Amato, Carlo Avolio, Paolo Gallo, Claudio Gasperini, Matilde Inglese, Girolama Alessandra Marfia, Francesco Patti

**Affiliations:** 1 Neurology Unit, IRCCS San Raffaele Scientific Institute, Via Olgettina 60, 20132 Milan, Italy; 2 Neurorehabilitation Unit, IRCCS San Raffaele Scientific Institute, Milan, Italy; 3 Neurophysiology Service, IRCCS San Raffaele Scientific Institute, Milan, Italy; 4 Neuroimaging Research Unit, Division of Neuroscience, IRCCS San Raffaele Scientific Institute, Milan, Italy; 5 Vita-Salute San Raffaele University, Milan, Italy; 6 University of Florence, Florence, Italy; 7 IRCCS Fondazione Don Carlo Gnocchi, Florence, Italy; 8 Department of Medical and Surgical Sciences, University of Foggia, Foggia, Italy; 9 Azienda Ospedaliero-Universitaria Policlinico, Foggia, Italy; 10 University of Padua, Padua, Italy; 11 Azienda Ospedaliera of Padua, Padua, Italy; 12 Azienda Ospedaliera San Camillo Forlanini, Rome, Italy; 13 Department of Neuroscience, Rehabilitation, Ophthalmology, Genetics, Maternal and Child Health (DINOGMI), University of Genoa, Genoa, Italy; 14 IRCCS Ospedale Policlinico San Martino, Genoa, Italy; 15 Multiple Sclerosis Clinical and Research Unit, Fondazione Policlinico Tor Vergata, Department of Systems Medicine, University Tor Vergata, Rome, Italy; 16 Department of Medical and Surgical Sciences and Advanced Technologies, GF Ingrassia, University of Catania, Catania, Italy; 17 Azienda Ospedaliero-Universitaria Policlinico “G. Rodolico-S. Marco”, Catania, Italy

**Keywords:** Bruton’s tyrosine kinase inhibitor, Italy, Multiple sclerosis, Progression independent of relapse activity, Relapse, Smouldering multiple sclerosis

## Abstract

The classification of multiple sclerosis (MS) into the two distinct phases of relapsing–remitting and progressive, including primary progressive and secondary progressive phenotypes (PPMS and SPMS, respectively) has long been accepted; however, there are several unmet needs associated with this particular model. The observation that both inflammation and neurodegeneration are present from the onset of MS has resulted in a paradigm shift towards MS as a disease continuum driven by pathological mechanisms underlying clinical progression. Here we report the results from a meeting of Italian MS specialists, exploring the evolving perception of MS pathobiology and its implications for diagnosis and treatment. Insights garnered from the expert panel advocate for a redefined understanding of MS. This expert opinion paper reviews the disease continuum and the intertwined nature of inflammatory and neurodegenerative processes. Also, the need for changes in diagnostic criteria and treatment strategies, including the development of novel biomarkers and new therapies targeting smouldering disease, is discussed.

**Supplementary Information:**

The online version contains supplementary material available at 10.1007/s00415-025-12917-4.

## Introduction

The earliest medical descriptions of multiple sclerosis (MS) date to the first half of the nineteenth century, with these descriptions already highlighting the relapsing–remitting nature of the disease [1]. In the subsequent decades, two classic clinical phenotypes of MS emerged: relapsing–remitting MS (RRMS) and progressive MS [2]. Classification of MS into the relapsing–remitting, primary progressive (PPMS), and secondary progressive (SPMS) phenotypes was formalised by the United States Advisory Committee on Clinical Trials of New Agents in Multiple Sclerosis for the National Multiple Sclerosis Society (NMSS) in 1996, with revisions following in 2014 [2, 3]. The objective of the NMSS classification was to provide clear and consistent definitions of patient groups [3]. The NMSS classification has been used in almost all clinical trials since its creation and has become widely adopted in clinical practice [3]; however, it is based solely on clinical course, and the authors noted at the time that there were no biomarkers for the various phenotypes [2].

The NMSS classification has been highly influential in shaping the management of MS. The majority of approved treatments for MS are indicated for the relapsing–remitting form of the disease and the NMSS classification is credited with the progress that has been made in the treatment of this form of MS [4]. However, there are few drugs that have been approved for progressive forms of MS [4], and despite the widespread use of the NMSS classification, accumulating evidence suggests that the nature of the underlying pathology is the same across all phenotypes [5, 6]. Furthermore, patients often experience disability accrual despite stable inflammatory parameters [7], highlighting a dissociation between focal inflammatory mechanisms and those accounting for the accumulation of disability. Thus, there is a need for a novel understanding of MS that reflects the current state of knowledge about its mechanisms [8].

This expert opinion paper describes the insights from a meeting of Italian MS experts and explores the evolving perception of MS pathobiology and its implications for diagnosis and treatment.

## Methods

The primary objective of this expert opinion paper was to analyse and synthesise the findings from an advisory board meeting held in Rome on 29 May 2023 (see the Supplementary Methods for more details). The findings and consensus from the advisory board were compiled and analysed to form the basis of this expert opinion.

## MS as a continuum

Traditionally, MS has been understood as progressing in two distinct phases [9]. The initial phase is characterised clinically by relapses and remissions, and biologically by active inflammation within the central nervous system (CNS). The subsequent phase is characterised by non-inflammatory neurodegeneration that manifests as progressive disability accumulation [9, 10]. However, a growing body of evidence suggests that this view is incorrect.

Recent studies have demonstrated that long-term disability accrual in MS cannot be reliably predicted based on the frequency of relapses or the presence of so-called “no evidence of disease activity” (NEDA), which includes the absence of relapses, magnetic resonance imaging (MRI) activity or disability accrual [11, 12]. Indeed, it is believed that irreversible accumulation of disability is caused by relapse-associated worsening (RAW; i.e. a step-wise increase in impairment due to incomplete recovery from relapses) during the early disease stages and progression independent of relapse activity (PIRA; i.e. relentless accumulation of permanent disability due to disease progression and ageing-related neurodegenerative processes), which becomes prominent in later stages [7, 13]. Moreover, therapeutic suppression of relapses in patients with RRMS does not necessarily prevent long-term disability accrual, with 65–90% of patients continuing to experience disability accumulation despite successful suppression of acute focal inflammation [14–16], indicating that relapses are not the sole drivers of this process [9, 15, 17]. This is further supported by observations of relapses overlapping with primary and secondary progressive courses in up to 40% of patients [18, 19] and similar clinical progression and disability levels in patients with SPMS and PPMS [20].

Mounting evidence suggests that both inflammation and neurodegeneration are present from the onset of MS, challenging the notion that these processes occur in separate disease phases. It is now clear that the pathological mechanisms underlying clinical progression begin early in the disease course [17]. MRI and pathological studies have shown neuroaxonal loss from the early stages, alongside active inflammation and demyelination [21–23]. This is consistent across different forms of MS, with pathology studies revealing similar levels of inflammatory infiltration, axonal loss and cortical demyelination in SPMS and PPMS [24–27].

A critical aspect of MS development is the occurrence of disability via PIRA. Studies have identified PIRA in patients early in the disease course, with an increased incidence after the fifth year, challenging the belief in a clear demarcation between inflammatory and neurodegenerative phases [28]. Furthermore, patients can exhibit disability accrual despite the absence of new or enlarging T2-weighted lesions or T1-weighted lesions, suggesting mechanisms of progression beyond inflammatory activity visible on MRI [5, 29]. Even in later stages of MS, including PPMS and advanced disease, inflammation remains a significant factor [30–32], and active inflammation and demyelination have been observed in the CNS of patients with terminal or end-stage MS [33]. The emerging consensus is that patients with MS of all clinical phenotypes have the same fundamental pathology, with both inflammatory and neurodegenerative processes occurring simultaneously [6, 34–37]. It is a new model for MS as a disease continuum, with varying clinical presentations believed to be due to differences in age, sex, genetics and other factors [38, 39].

## Smouldering disease

Smouldering disease is an umbrella term that refers to the chronic pathobiological processes occurring in the CNS, other than focal inflammation, associated with neurodegeneration, leading to clinical worsening in people with MS [40, 41]. Smouldering disease begins early, before the development of clinical symptoms [5], and can be identified histopathologically by the presence of myelin breakdown products in macrophages and microglia at the borders of lesions and, more recently, by MRI [17, 42].

In patients with MS, smouldering disease is distinct from PIRA and should not be confused with it [43]. PIRA refers specifically to clinical manifestations as measured using the Expanded Disability Status Scale (EDSS) [43]; however, it does not provide the full picture and has certain limitations (e.g. it does not address the biology), which prevents it from being widely used in clinical practice. Smouldering disease, on the other hand, attempts to overcome these limitations by including other markers and manifestations of end-organ damage, such as whole brain volume loss, grey matter loss, thalamic atrophy, chronic active lesions (CALs), increased neurofilament levels, cognitive decline and retinal nerve fibre layer thinning, as well as declining performance on neurological stress tests, such as walking distance, running speed, hand–eye coordination and balance tests [43]. In addition, other promising biomarkers include optical coherence tomography (OCT) and blood biomarkers linked to compartmentalised inflammation (e.g., fluid biomarkers of PIRA) and neurodegeneration (e.g., synaptic pathology) [17, 44, 45]. Also, it is important to note that disease-modifying therapies (DMTs) can affect PIRA [28], which is one of the manifestations of smouldering disease.

CALs are MS lesions characterised by persistent low-level inflammation, axonal damage, demyelination and expansion [26, 46–48]. CALs constitute 20–40% of white matter lesions [5, 49]. Active demyelinating lesions, present in smouldering disease, consist of a core of CD8 + T lymphocytes and CD20 + B lymphocytes, as well as a small number of plasma cells, surrounded by an outer rim of iron-laden activated microglia, macrophages and oligodendrocytes [5, 17, 49–51]. The iron found within the microglia forms a paramagnetic rim that can be visualised using susceptibility-weighted imaging (SWI) techniques [52–54]. The number of paramagnetic rim lesions increases over the course of MS [26] and correlates with cognitive impairment, disability and progression in both RRMS and progressive MS [47, 55–59].

Microglia are the resident phagocytes of the CNS, where they are involved in immunological surveillance and homeostasis maintenance [60]. The functions of microglia include phagocytosis of myelin debris, production of growth factors, reshaping of neuronal circuits and assisting in re-myelination [60]. Microglia appear to play an important role in the development and evolution of MS, particularly in smouldering disease. Activated microglia are the most common myeloid cells found in the active lesions in patients with progressive MS [61], dominating the outer rims of paramagnetic rim lesions [62]. Activated microglia are found throughout the normal-appearing white matter of patients with MS [32]. Early in the disease course, nodules of activated microglia form in the normal-appearing brain matter of patients with MS and can subsequently evolve into lesions [63–66]. Activation of microglia and/or macrophages has been associated with the development of disability in patients with RRMS and SPMS, even if they had no relapses [67]. Whilst an attractive therapeutic target, currently available DMTs have modest effects on microglia due both the lack of a specific target and an inability to reach adequate concentrations in the CNS [68].

Identifying the transition from the RRMS to the SPMS phenotypes in the classically accepted MS model currently represents a significant challenge for clinicians [69–71]. There are no standard criteria for detecting this transition, and clinical signs and symptoms vary amongst patients [69, 71]. Diagnosis of SPMS is often made at the discretion of the treating physician, taking into account factors such as worsening physical disability independent of relapses, cognitive decline and the onset of persistent symptoms [69]. As a result, SPMS is often diagnosed retrospectively, with a delay of up to 5 years [71, 72].

Several authors have proposed objective clinical criteria for identifying the transition from the RRMS to the SPMS phenotype [69, 73], but with a lack of consensus among research groups. A set of criteria based on the change in EDSS scores has been shown to reduce the diagnostic delay by over 3 years [73]. However, this definition is hampered by its reliance on EDSS as the sole measure of functional decline [69]. From a biological perspective, development of diffuse microglial activation and neurodegeneration become more common in SPMS than in RRMS [74]. The Italian MS Registry recently compared the diagnostic performances of two different data-driven SPMS definitions based on a version of Lorscheider’s algorithm (DDA) and on the EXPAND trial inclusion criteria, using the neurologist’s definition (ND) as gold standard [75]. This comparison revealed that data-driven definitions are more adept than the ND definition at capturing SP transition, and the global accuracy of DDA appears to be higher than the EXPAND definition. The difficulty in slowing down or improving disability in progressive MS highlights the importance of early treatment aimed at prompt prevention of disability accumulation.

Given their critical role in immunological processes as specialised monocytes in the CNS [76], microglia represent the obvious choice as a novel and key target for the development of new drugs for the prevention of disability accrual in MS. However, microglia should not be considered the only target, as peripheral monocytes and lymphocytes, including CD20-expressing cells, also provide promising targets of smouldering disease, as well as other glial cells (e.g. [reactive] astrocytes, oligodendrocytes) and their precursors (failed re-myelination). The possibility of neuroprotective strategies targeting stressed neurons should also be considered as a complementary approach to prevent disability accrual; with complex interplay between the cells, acting on one could indirectly affect the others. Although other drugs have also shown effects on microglia in in-vitro studies (for reviews of treatment, see Refs. [77–82]), action on microglia is not thought to be one of the main mechanisms of action of currently available drugs. Therefore, here we focus on the new class of Bruton’s tyrosine kinase (BTK) inhibitors, since these agents represent the first to act primarily on microglia. Thus, it could be speculated that additional effects on disability progression are due to the action of BTK inhibitors on compartmentalised inflammation.

BTK is a non-receptor tyrosine kinase expressed in a number of adaptive immune cells, including B cells, and innate immune cells, including macrophages and microglia, where it plays crucial roles in the activation and release of pro-inflammatory cytokines [16]. Thus, BTK inhibitors have the potential to address biological and clinical manifestations of smouldering disease, hopefully leading to reduced disability accumulation, which remains the unmet need in MS.

Several BTK inhibitors are currently being developed for the treatment of MS [16]. Their mechanism of action and the ability to cross the blood–brain barrier have the potential to broadly impact the biological mechanisms underlying the disease as previously discussed. In particular, tolebrutinib is currently in clinical development across the entire spectrum of the disease: GEMINI 1 and 2 trials (NCT04410978, NCT04410991) in relapsing forms of MS, the HERCULES trial (NCT04411641) in non-relapsing SPMS, and the PERSEUS trial (NCT04458051) in PPMS. Results of these phase III studies provide further confirmation of the common biological mechanisms underlying the different forms of MS. Recently presented results of the HERCULES study showed that tolebrutinib significantly reduced disability accumulation compared with placebo in patients with non-relapsing SPMS, with a 31% reduction in the time to 6-month confirmed disease progression (*p* = 0.0026), an increased proportion of patients achieving confirmed disability improvement (10% vs 5%; *p* = 0.021), and significantly reduced annualised rate of new/enlarging T2 lesions (adjusted rate ratio, 0.62; 95% confidence interval 0.43, 0.90; *p* = 0.011) [83]. Contrastingly, results of the GEMINI 1 and 2 studies showed no significant difference in the primary endpoint of annualised relapse rate with tolebrutinib versus teriflunomide, despite a significant pooled 29% reduction in confirmed disability worsening with tolebrutinib (*p* = 0.23) [84]. Whilst failure to show a statistically significant difference in the primary endpoint between treatment groups might be perceived as disappointing, it is important to remember that the annualised relapse rate was very low overall and the study included an active comparator; also, the study was powered to detect disability. Indeed, the fact that disability was reduced with tolebrutinib despite with such low rates of relapse might suggest that progression is driven by mechanisms independent of relapses.

Similarly, phase III studies of evobrutinib did not show superior effects on annualised relapse rate over teriflunomide in patients with RRMS, but also failed to show superiority in reducing disability [85]. These differences in efficacy outcomes between tolebrutinib and evobrutinib may be due to the different properties of the two molecules, and the reduced ability of evobrutinib to cross the blood–brain barrier compared with tolebrutinib [86].

## Expert opinion

**Fig. 1:**
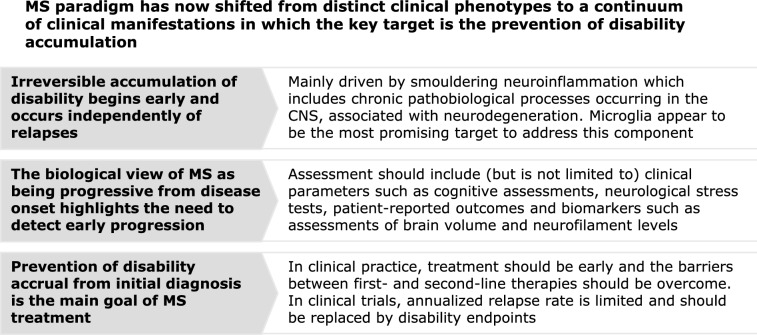
The multiple sclerosis paradigm has shifted from distinct clinical phenotypes to a continuum of clinical manifestations in which the key target is the prevention of disability accumulation. *CNS* central nervous system, *MS* multiple sclerosis

During the advisory board meeting, participating experts endorsed the paradigm shift in the understanding of MS (Fig. 1). Accumulated evidence supports a move away from a view of MS as a collection of distinct clinical phenotypes to the novel understanding of a continuum of clinical manifestations explained by the same underlying biological mechanisms. This shift has been facilitated by a change in focus from macroscopic inflammatory changes visible on MRI to microscopic alterations described in histological studies. Smouldering disease, characterised by compartmentalised chronic neuroinflammation, with involvement of microglia, macrophages, and lymphocytes, leading to neurodegeneration and disability accrual [17, 50, 51], is known to manifest early and across the spectrum of MS [47, 56]. A recent investigation into the historically identified clinical MS phenotypes found no qualitative differences amongst them regarding multiple pathological features associated with clinical progression, which appears to support the contemporary view that MS in one disease in which several types of events can occur (i.e., attacks and progression), as opposed to distinct clinical entities [87].

This change in perspective is expected to have significant implications for the diagnosis and management of MS. The biological view that all forms of MS should be considered active and progressive, starting from disease onset, highlights the acute need for ways to detect early progression. Whilst traditionally used methods, such as cognitive assessments, neurological stress testing, and in-depth consultation, will continue to play an important role in the assessment of patients, new markers for the surveillance of disease progression are needed. Patient-reported outcomes (PROs) hold significant promise due to their ability to detect early signs of neurodegeneration [88], as well as the accessibility to relevant measures and low cost to administer. However, the experts pointed out several challenges associated with the use of PROs, namely the need for extensive validation, challenges in widespread implementation and, in some cases, poor adherence. Brain volume, neurofilament level and OCT assessments are expected to be incorporated into clinical practice and to contribute to individualised care [5, 45, 89]. However, it is important to note that the use of neurofilament level has some limitations as a sole biomarker, namely association with active inflammation rather than neurodegeneration [90]. Participating experts highlighted the complimentary nature of traditional and novel assessments and biomarkers in facilitating the diagnosis and follow-up of patients with MS, and the need for further biomarker development to capture the complexity of MS, including markers of compartmentalised inflammation, de-/re-myelination and neurodegeneration.

The experts agreed that the goal of MS treatment from initial diagnosis should be prevention of disability accrual. EDSS is able to detect those patients with PIRA at the first demyelinating attack [91]. The annualised relapse rate represents a limited primary endpoint for MS pharmacological trials and should be replaced by disability endpoints. Furthermore, considering that acute inflammation is well controlled by current DMTs resulting in low annualised relapse rates, new targeted drugs are unlikely to further reduce this, thus the endpoint could become a barrier to the development and introduction of novel targeted therapies into clinical practice. In this respect, if the GEMINI studies had utilised disability as the primary outcome measure, the trials would have given positive results representing a solid evidence base for regulatory authorities. Moreover, the shift towards a biological understanding of MS suggests that, for prescriptive purposes, the distinction between first- and second-line therapies should be abolished, with the experts, instead, emphasising the importance of early treatment. Regulators and payers should adopt the biological understanding of MS to ensure patients receive access to effective treatments before the development of disability.

It is now evident that treatment must be initiated as early as possible and with drugs that have the best evidence of effects on the progression of disability. The wide range of drugs available allows tailored therapeutic choices with consideration of individualised benefit/risk profile, inflammatory burden, the presence of prognostic factors and patient compliance. In addition, the availability of new options for non-relapsing SPMS will open up new sequencing and treatment opportunities for a de facto orphan population.

## Conclusion

The insights garnered from the expert panel advocate for a redefined understanding of MS, emphasising the disease’s continuum and the intertwined nature of inflammatory and neurodegenerative processes. This perspective necessitates a reassessment of diagnostic criteria, treatment strategies and patient care approaches, paving the way for more effective management of all forms of MS. It is hoped that novel therapies, such as BTK inhibitors with the ability to target smouldering disease, may help to prevent disability accrual from early in the disease course. As the field moves forward, it is crucial that clinical practice and research align with this evolving understanding to improve outcomes for individuals living with MS.

## Supplementary Information

Below is the link to the electronic supplementary material.

Supplementary file1 (DOCX 27 KB)
