# Supplementary material for: Towards a biological view of multiple sclerosis from early subtle to clinical progression: an expert opinion
Source: J Neurol. 2025 Feb 1;272(2):179. doi: 10.1007/s00415-025-12917-4 (PMC11787267; doi:10.1007/s00415-025-12917-4)
Supplement: Supplementary file 1 — Supplementary file1 (DOCX 27 KB) [file 415_2025_12917_MOESM1_ESM.docx]

**Towards a biological view of multiple sclerosis from early subtle to clinical progression: an expert opinion**

**Authors:** Massimo Filippi, Maria Pia Amato, Carlo Avolio, Paolo Gallo, Claudio Gasperini, Matilde Inglese, Girolama Alessandra Marfia, Francesco Patti

**Supplementary Information**

# Supplementary Methods

## Details of the advisory board meeting

The meeting focused on the transition from classifying multiple sclerosis (MS) into several phenotypes based on clinical course to a perspective where MS is considered a continuum based on the underlying pathological mechanisms. The advisory board consisted of renowned MS experts from leading Italian universities and hospitals. Presentations and discussions were structured around key questions regarding the disease continuum, the definition of smouldering disease and the clinical relevance of various pathological components and treatment approaches (see below), with a comprehensive review of current evidence on each of these three topics. To provide additional information in the current review, a PubMed search was completed in March 2024 (see below) and relevant publications were suggested by the meeting participants. The findings and consensus from the advisory board were compiled and analysed to form the basis of this expert opinion.

## Discussion questions

| Smouldering MS: definition, key components, clinical and instrumental assessments, implications for treatment, current cultural gap |
| --- |
| - Is MS a continuum? - Do you agree with the definition of smouldering MS? - Is the smouldering component always present in the course of the disease? And how important is it compared to the other components? - How relevant is the role of microglia and compartmentalised inflammation? - What lesions and what other radiologically observable phenomena are most relevant? Are there other types of potentially useful instrumental measurements? - How is it possible to measure smouldering from a clinical point of view? - PIRA: is it sufficient to define the progression of disability associated with smouldering? - Does the concept of smouldering have implications for the way we think about the therapeutic approach? - In order for the concept of smouldering to be correctly understood by the medical-scientific community, how can we devise a correct and effective communication plan? What do you think are the current knowledge gaps and how can they be filled? |
| Progressive MS: definition, epidemiology and patient journey, unmet needs related to therapy |
| - To date, how is progression defined and measured? How could the ability to identify progression be improved? - Is the established classification of the disease as RRMS, PPMS and SPMS still clinically and therapeutically relevant? From a regulatory point of view, would it be appropriate to overcome it? - SPMS: Italian epidemiological data show variable results based on the different definitions, how to interpret them correctly? What is the proportion of nrSPMS patients? - What are the unmet progression-related needs related to the currently approved disease-modifying therapies? What are the strengths? |
| Tolebrutinib: therapeutic potential of BTK inhibition and relevance of central and peripheral effects, advantages and positioning compared to current therapies, differentiation of BTK inhibitors in development, how to effectively communicate the role of BTK inhibitors to the scientific community |
| - What are the strengths and therapeutic potential of BTK inhibition?   - Ability to enter the CNS, exert a central effect on CNS-resident innate immunity cells, inhibit activation and modulate microglia?   - Peripheral effect on B cells, through their modulation and not depletion?   - What key elements do you suggest to communicate the role of BTK inhibition to the scientific community? - How does tolebrutinib compare to currently approved disease-modifying therapies? - How to differentiate it within the class? - How should tolebrutinib be positioned to provide maximum benefit to patients (naïve, treatment-experienced, special populations)? - What is the relevance of pharmacological differences (e.g., half-life, inhibitory concentration) and clinical trial schedules of developing BTK inhibitors? - What would the clinical practical benefit be of a drug potentially indicated in relapsing MS, nrSPMS and PPMS? |

BTK, Bruton’s tyrosine kinase; CNS, central nervous system; MS, multiple sclerosis; nrSPMS, non-relapsing secondary progressive multiple sclerosis; PIRA, progression independent of relapse activity; PPMS, primary progressive multiple sclerosis; RRMS, relapsing-remitting multiple sclerosis; SPMS, secondary progressive multiple sclerosis.

## Literature search strategy

The PubMed database was searched using the following string:

("Multiple Sclerosis"[Mesh] OR "Multiple Sclerosis, Chronic Progressive"[Mesh] OR "Neurodegenerative Diseases"[Mesh]) AND ("Biomarkers"[Mesh] OR "Magnetic Resonance Imaging"[Mesh]) AND ("Therapeutics"[Mesh] OR "Treatment Outcome"[Mesh]) AND ("Diagnosis"[Mesh] OR "Diagnostic Imaging"[Mesh] OR "Diagnostic Techniques, Neurological"[Mesh]) AND "2020/01/01"[PDAT] : "3000"[PDAT]
